# Exploitation of cantaloupe peels for bacterial cellulose production and functionalization with green synthesized Copper oxide nanoparticles for diverse biological applications

**DOI:** 10.1038/s41598-022-23952-w

**Published:** 2022-11-10

**Authors:** Ahmed K. Saleh, Hamada El-Gendi, Esmail M. El-Fakharany, Medhat E. Owda, Mohamed A. Awad, Elbadawy A. Kamoun

**Affiliations:** 1 Cellulose and Paper Department, National Research Centre, El-Tahrir St., Post 12622, Dokki, Giza Egypt; 2 Bioprocess Development Department, Genetic Engineering and Biotechnology Research Institute, City of Scientific Research and Technological Applications (SRTA-City), New Borg El-Arab City, 21934 Alexandria Egypt; 3 Protein Research Department, Genetic Engineering and Biotechnology Research Institute, City of Scientific Research and Technological Applications (SRTA-City), New Borg El-Arab City, 21934 Alexandria Egypt; 4 Chemistry Department, Faculty of Science, Al-Azhar University, Nasr City, 11884 Cairo Egypt; 5 Zoology and Entomology Department, Faculty of Science, Al-Azhar University, Nasr City, Cairo Egypt; 6 Nanotechnology Research Center (NTRC), The British University in Egypt (BUE), El-Sherouk City, 11837 Cairo Egypt; 7 Polymeric Materials Research Department, Advanced Technology and New Materials Research Institute (ATNMRI), City of Scientific Research and Technological Applications (SRTA-City), New Borg El-Arab City, 21934 Alexandria Egypt

**Keywords:** Biological techniques, Biotechnology, Microbiology

## Abstract

The promising features of most bacterial celluloses (BC) promote the continuous mining for a cost-effective production approach toward wide and sustainable applications. Herein, cantaloupe peels (CP) were successfully implemented for sustainable BC production. Results indicated that the enzymatically hydrolyzed CP supported the maximum BC production of approximately 3.49 g/L when used as a sole fermentation media. The produced BC was fabricated with polyvinyl alcohol (PVA) and chitosan (Ch), and loaded with green synthesized copper oxide nanoparticles (CuO-NPs) to improve its biological activity. The novel composite showed an antimicrobial activity against several human pathogens such as *Staphylococcus aureus, Streptococcus mutans, Salmonella typhimurium, Escherichia coli,* and *Pseudomonas fluorescens*. Furthermore, the new composite revealed a significant in vitro anticancer activity against colon (Caco-2), hepatocellular (HepG-2), and breast (MDA) cancer cells, with low IC_50_ of 0.48, 0.27, and 0.33 mg/mL for the three cell lines, respectively. On the other hand, the new composite was remarkably safe for human skin fibroblast (HSF) with IC_50_ of 1.08 mg/mL. Interestingly, the composite membranes exhibited lethal effects against all stages of larval instar and pupal stage compared with the control. In this study, we first report the diverse potential applications of BC/PVA/Ch/CuO-NPs composites based on green synthesized CuO-NPs and sustainably produced BC membrane.

## Introduction

Cellulose is an important and abundant renewable biopolymer on earth. It is a polysaccharide formed by D-glucose units linked by β (1 → 4) glycosidic linkage^1^, retrieved primarily from plant cells, and produced by microbial fermentation^2^. Gram-negative bacteria of the family Acetobacteraceae, particularly the *Komagataeibacter* genus, (formerly known as *Gluconacetobacter*), are the main BC producer in commercial scales^3–5^. Gram-positive bacteria were also reported for BC production through some species^6–8^. BC is an extracellular polysaccharide that exhibits distinctive properties such as high water retention capacity, degree of crystallinity, mechanical strength, formability, hydrophilicity, biocompatibility, flexibility, and nontoxicity^9,10^.

According to these excellent properties, BC has been applied in different fields including biomedical^11^, cosmetics^12^, and food industry^13^**.** However, BC production is still challenging, attributed to the high cost involved and strains of low productivity. Fermentation media was account for one/third of the total production cost, hence digging for cost-effective and easily available carbon sources could improve the production process^14^. Several renewable biomass, such as fruit peels, are widely available in large quantities and have been reported as a low-cost-effective media for BC production, such as orange^15^, cucumbers, melon, kiwifruit, apple, and quince peels^3^, pineapple^16^, and banana^17^.

Cantaloupe is a popular fruit worldwide because of its delicious aroma and nutritive value. It is also an excellent source of vitamins (A and C), β-carotene, polyphenol antioxidants, and microelements such as potassium and magnesium, which provide important health benefits and decrease the risk of chronic diseases^18–20^. The processing of cantaloupe or melon fruit juice, syrup, compotes, salads, and jam produces a significant amount of outer rind (peels) as a by-product, which represents a considerable environmental challenge and necessitates a biorefinery operation rather than being regarded as negligible waste and disposed^21^.

Previously, several researchers used CP to produces enzymes such as alpha amylase^22^, xylanase^23^, and laccase^24^. Nevertheless, BC is a biologically and naturally synthesized biomaterial, and there are numerous attempts to modify and functionalize BC using either in situ or ex situ methods to produce a polymeric composite with distinctive applications. BC modification with hyaluronic acid (HA)^25^, sodium alginate^26^, graphene oxide^27^, starch^28^**,** chitosan^29^ and metal nanoparticles^30^ was widely reported with enhanced membrane characteristics. Recently, the loading of BC with different nanometals has emerged as an effective approach to increase the scope of BC application and lower nanometal toxicity^31,32^.

Among the materials, CuO-NPs are biocompatible and rarely toxic, which promotes their application potential^33,34^. Two approaches were applied for the synthesis of nanoparticles, including physicochemical and green approaches. Green synthesis of nanoparticles depends on the use of ecofriendly reducing enzymes and stabilizing agents from microbial or plant extracts. Green CuO-NPs are synthesized from plant extract^35,36^, bacteria^37,38^, actinomycetes^39,40^, fungi^41,42^, and algae^43,44^. At present, CuO-NPs are widely reported for different medical applications because of their affordable price (compared with silver and gold-NPs) and diverse biological activities, however, the stability of particles remains a key challenge^45^. The high energy of the NP surface causes the CuO-NPs free particles to aggregate into a larger cluster and induce NPs precipitation^46^. This precipitation is usually associated with the loss of a biological activity, hence loading onto supporting membranes can sustain the biological activity. Additionally, the slow and controlled release from the supporting membrane could alleviate the potential overdose toxicity from the applied metals^47^. Recently, CuO-NPs are fabricated for versatile applications for example, chitosan (BC/ chitosan) for dentistry application^48^, graphene oxide (BC/graphene oxide) for antibacterial activity^49,50^, polyethylene oxide/polyvinyl pyrrolidone for electrical conductivity^51^, and titanium oxide nanoparticles for photocatalytic degradation^52^.

**Figure 1:**
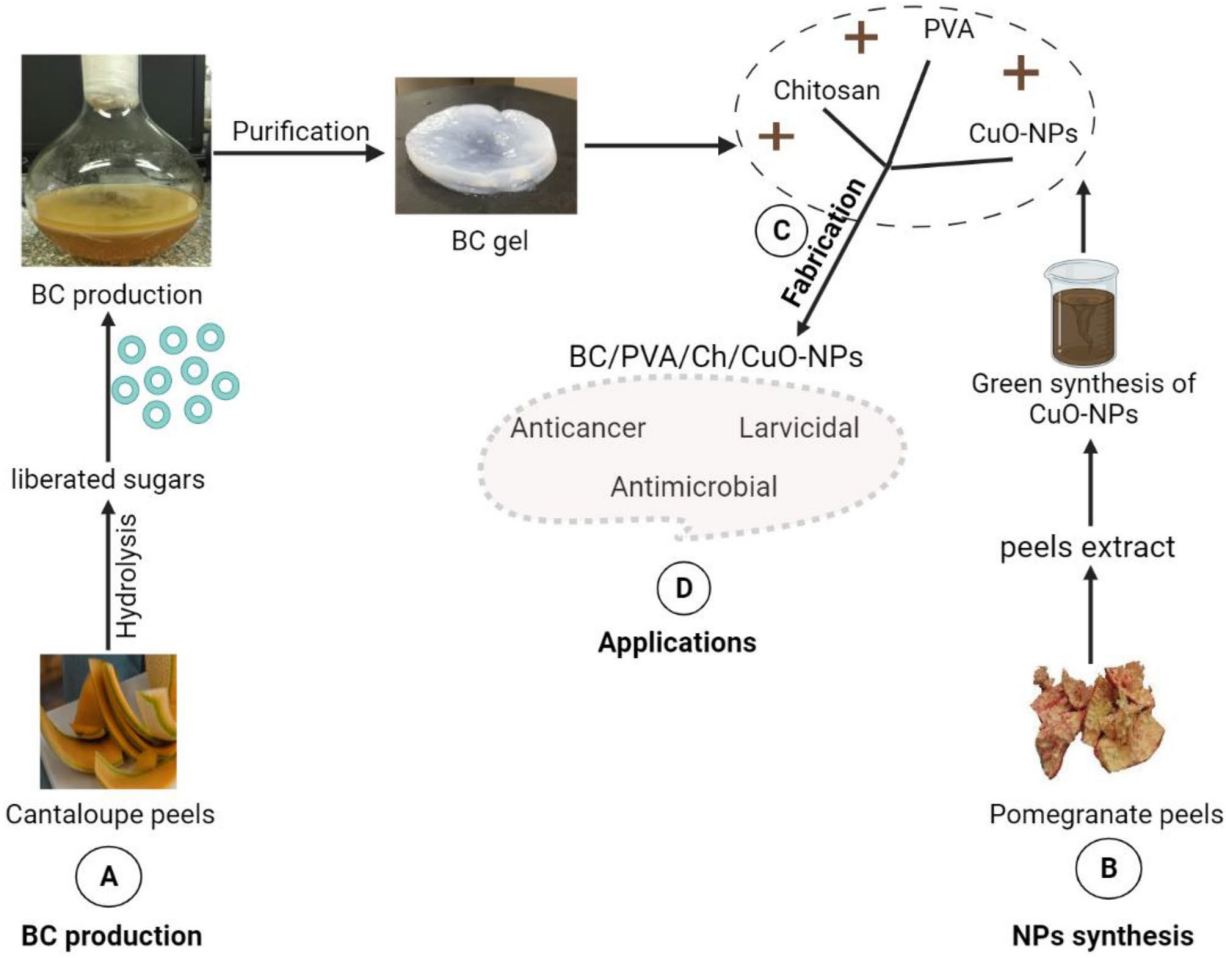
Schematic representation of the four main steps in the present study: (**A**) synthesis of BC using CP media, (**B**) green synthesis of CuO-NPs using POP extract, (**C**) fabrication of the resulting BC membrane with PVA and Ch, and (**D**) potential composite applications (**D**).

Among other medical applications, the implementation of CuO-NPs in antimicrobial and anticancer treatment is gaining considerable attention^53^. The lack of an effective cancer treatment hinders the search for novel high-selectivity treatments^54,55^. In addition, current cancer treatment strategies usually result in immune suppression in treated patients, which increases the risk of microbial infection^56^. Therefore, finding novel anticancer agents with an antimicrobial activity is necessary to alleviate treatment complications. Hence, the augmentation of BC with CuO-NPs could result in a scaffold for several bioactivities and diverse applications. In this work, we first reported the application of enzymatically hydrolyzed cantaloupe peel (ECP) as a low-cost media for sustainable BC production. In addition, the biosynthesis of CuO-NPs using pomegranate peel (POP) extract was reported. The preparation of a composite membrane (BC/PVA/Ch) loaded with green synthesized CuO-NPs as a scaffold for diverse biological activities (Fig. 1) was also evaluated and characterized.

## Results and discussions

### Saccharification of CP for BC production

**Figure 2:**
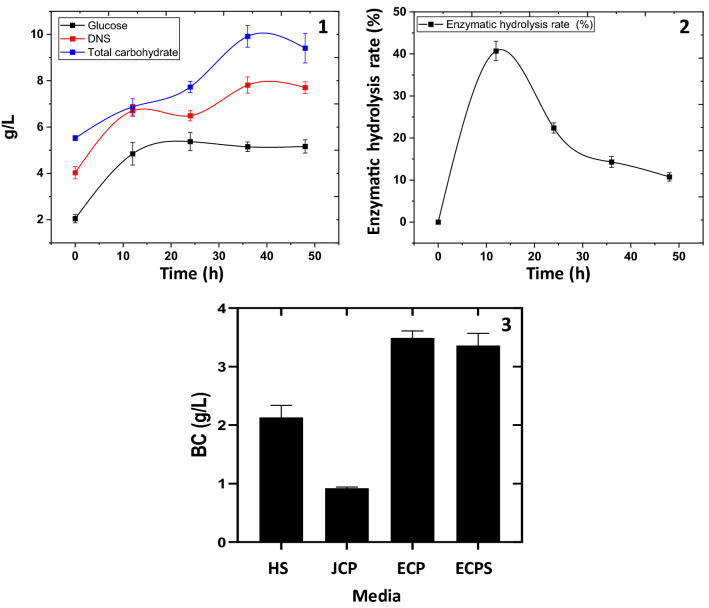
Scarification of CP (1), enzymatic hydrolysis rate (2) at different time intervals, and BC production from different media (3).

The rate and cost of BC production were directly influenced by the availability of the origin and type of carbon sources in the fermentation media^57–59^. To increase the content of fermentable sugars, the fresh CP was initially milled through a high-speed blender prior to enzymatic hydrolysis using cellulases. The results (Fig. 2(1)) indicated a considerable amount of total carbohydrates in the fresh CP (5.52 g/L) with initial glucose and reducing sugar contents of about 2.04 and 4.03 g/L, respectively. This small amount of glucose (2.04 g/L) in the fresh CP could be attributed to the peeling process, where small parts of the cantaloupe flesh remain in the peels. During the saccharification process, a high value of total carbohydrate content (9.91 g/L) was observed after 36 h of enzymatic hydrolysis. The initial pre-treatment of CP with milling increases the accessibility of cellulases to plant fibers and hence higher carbohydrate content was available, which is in accordance with Akintunde et al.^60^, and Kucharska et al.^61^. Cellulase hydrolyzes the 1,4-beta-D-glucosidic linkage in the cellulose structure, liberating beta-glucose, shorter polysaccharides, and oligosaccharides depending on the plant waste structure as stated by Bayitse et al.^62^. Application of cellulases to the fresh CP significantly elevated the glucose and total reducing sugar to a maximum of 5.372 g/L glucose after 24 h and 7.8 g/L reducing sugar after 36 h of hydrolysis. The maximum sugar generated through enzymatic hydrolysis represents 2.63- and 1.94-fold increases in glucose and reducing sugar, respectively, compared to their initial concentrations before hydrolysis. Furthermore, the results asserted the efficiency of the milling and enzymatic hydrolysis processes for CP saccharification as the maximum content of carbohydrate was 9.92 g/L (after 36 h), where total reducing sugar was 7.81 g/L after the same time, indicating that only about 2 g/L of the original carbohydrate was not subjected to hydrolysis. On the other hand, as show in Fig. 2(2), the enzymatic hydrolysis rate of CP decreased with the time of hydrolysis increased. Though several studies have found that synthetic carbon sources such as glucose, mannitol, sucrose, and fructose have a high potency in increasing BC production yields ^63–66^. The high cost involved forced application of agricultural and/or industrial wastes as a cost-effective alternative for BC production, including potato peels, tobacco waste, and starch kitchen wastes ^67–69^.

In reducing the cost of feedstock for BC production, CP was used to prepare media as an alternative to the commonly used, but expensive, Hestrin and Schramm (HS) media. Four different media were applied namely HS, JCP, ECPS, and ECP, for BC production by using *L. plantarum* AS.6. As shown in Fig. 2(3), after 7 days of static cultivation, the BC production from JCP medium was 0.92 g/L; this result is lower than that obtained from HS media (2.13 g/L). The enzymatic hydrolysis of CP was performed to enhance the BC production yield from ECP and ECPS which reached 3.49 and 3.36 g/L, respectively. Based on our results, the highest BC production was obtained from ECP, followed by ECPS, and then the lowest production was observed in JCP. High BC yields (3.8 and 1.6 fold) were obtained in enzymatic hydrolysate cultures compared with that achieved by *L. plantarum* AS.6 in JCP and standard HS media, respectively. This result is consistent with that of other study that is, the enzymatic hydrolysis of orange peels enhances BC production, which is1.4 fold higher than that in orange peels without hydrolysis^70^. Another study has reported that higher BC yields were obtained in cotton enzymatic hydrolysate cultures (1.8 folds) compared with that achieved in glucose-based cultures^71^. Wang et al., reported that the BC yield from enzymatic hydrolysis of sweet sorghum (root, stalk, and leaf) was 1.86–2.59-fold higher than the glucose-based BC yield^72^. The results of this study were also compared favorably with these published works. The results indicate the importance of hydrolysis in releasing simple sugars from complex polysaccharides, increasing their suitability for microbial fermentation and enhancing the BC production rate. Moreover, the complex components of CP, could promote the formation of BC. The production rate of BC from different media was 0.30, 0.13, 0.48, and 0.50 g/L/d from HS, JCP, ECPS, and ECP media, respectively. The results showed that adding glucose-free HS media to ECP has no effect (not significant) on BC production when compared with sole ECP. Several studies have found that enzymatic hydrolysis of waste promotes BC production compared with conventional culture media, which is consistent with the current results presented in Table 1. Therefore, comparing the published data with the current study, the ECP media could be considered as a potentially low-cost and high-yield media for obtaining BC.

**Table 1:** Comparison of BC production using enzymatic hydrolysis of different wastes.

Waste type	Strain	Enzyme	BC g/L from standard media	BC g/L from waste hydrolysate media	Reference
Cantaloupe peel	*L. plantarum* AS.6	(89.4 FPU) cellulase	2.13	3.49	Current study
Orange Peel	*Gluconacetobacter xylinus*	(30,000 U) Cellulase and (1200 U) pectinase	0.97	6.1	Kuo et al.^70^
Oat hulls	*Medusomyces gisevii* Sa-12	(25 FPU) CelloLux-A and (15 FPU) BrewZyme BGX	ND	2.2	Skiba et al.^73^
Sugarcane straw	*Komagataeibacter xylinus* ATCC 11,142	(240 FPU) Cellic® CTec2	1.06	4.51	Dhar et al.^74^
Sweet sorghum	*Acetobacter xylinum* ATCC 23,767	(20 FPU) Cellic® CTec2	0.98	2.54	Wang et al.^72^
Caragana	*Gluconacetobacter xylinus* CGMCC 2955	(15 FPU) Cellic CTec2 and (9 U) Cellic HTec2	3	4.6	Li et al.^75^
Citrus pulp water	*Gluconacetobacter xylinum*	(50 U) cellulase and (150 U) pectinase	1.6	8.77	Cao et al.^76^
Coconut water				9.91	
Cheese whey	*Gluconacetobacter* *xylinus* PTCC 1734	Lactase	3.26	3.55	Salari et al.^77^
Grains and yellow water from Baijiu production	*Gluconacetobacter* *xylinus* G29	Cellulase and Glucoamylase	0.86	7.42	He et al.^78^
Starch kitchen wastes	*Komagataeibacter hansenii AS.5*	(313 U) Amylase	ND	2.11	Saleh et al.^69^
Colored rice	*Komagataeibacter xylinus* AGR 60	(300 U) Starch degrading enzyme and (2 U) Glucoamylase	ND	8.15	Noree et al.^79^

### Characterization of BC membranes

#### SEM investigation

**Figure 3:**
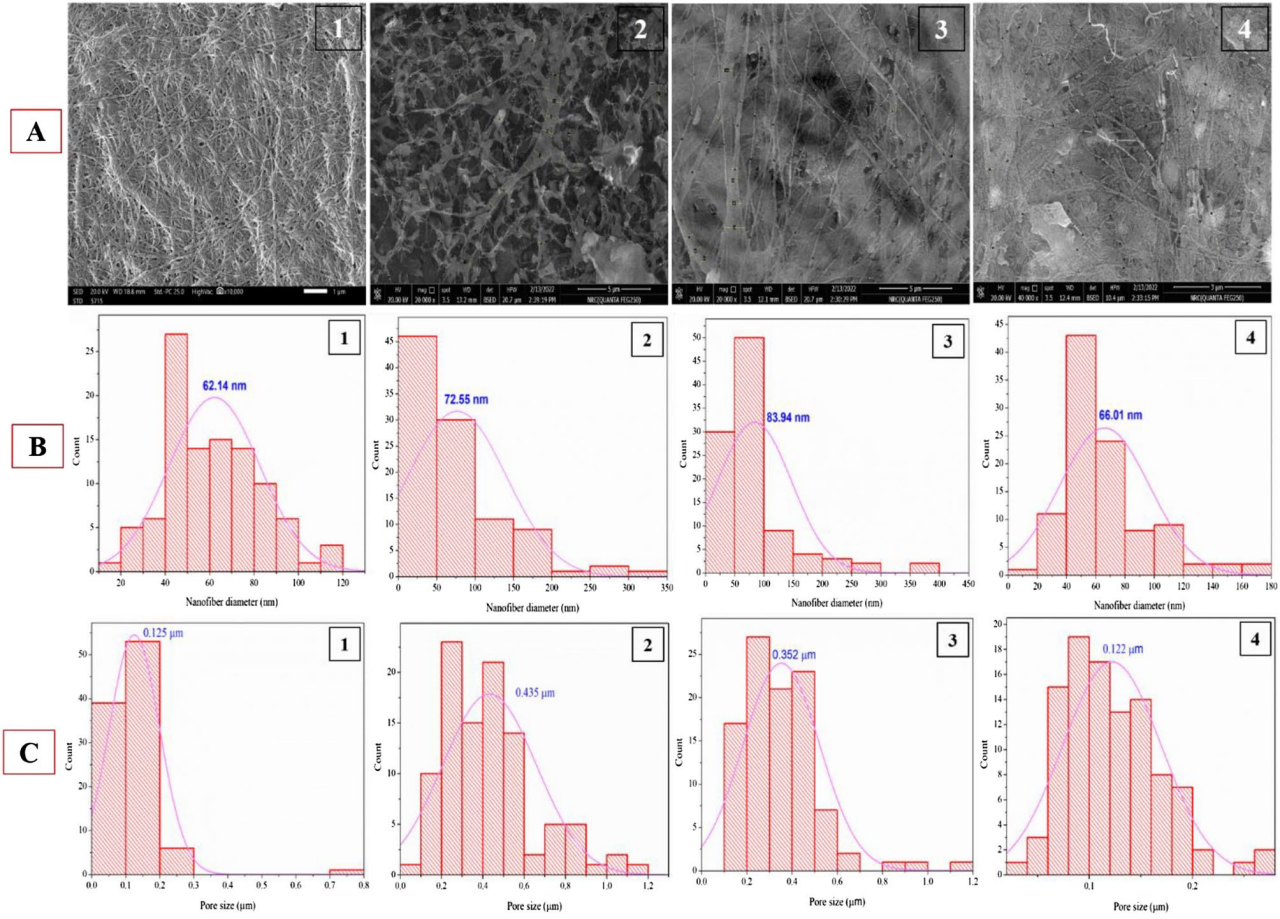
SEM micrographs of BC membranes (**A**), nanofiber diameter (**B**), and pore size diameter (**C**) using different cultivation regimes; HS (1), JCP (2), ECPS (3), and EPC (4) media where all images of SEM were taken with (scales 1, 5 µm, and original magnification 20,000X with applied voltage of 20 kV).

Figure 3A, B and C shows the surface morphological structure, nanofiber and pore size distribution of BC derived from HS, JCP, ECPS, and ECP media. The surface morphology of BC obtained from HS media shows dense, interconnected, homogeneous, and random oriented nanofibers with an average spore size of 0.121 µm. It is also clear that the randomly oriented nanofibers and the fibrillary shape-structure didn’t exhibit empty spaces between BC nanofibers, and the average nanofiber distribution is 62.14 nm. However, BC derived from JCP media shows enlarged spaces between nanofibers with a low density, which may suggest high porosity. The diameters of the pores and the average nanofiber distribution are 0.468 µm and 72.55 nm, respectively. The BC obtained from ECPS and ECP media showed significant similarity with very loose fibril surface structure and an irregular, fluffy interior structure. The average nanofiber distribution and pore sizes were (83.94 and 66.01 nm) and (0.334 and 0.111 µm) for BC obtained from ECPS and ECP media, respectively. The average nanofiber diameter and pore size of BC membranes were calculated by measuring around 50–100 points per sample. The network morphology of the BC based on CP media was slightly different from that of the BC derived from SH media. According to these observations, the morphological structure and nanofiber distribution differed according to the fermentation conditions (static or agitated), producer strain, and biomass sources, and these results are consistent with other reported studies^76,80–82^. These distinctions can have an impact on the final application of obtained BC, as either porous or compacted surface structures are important when creating a specific device or environment^83,84^.

#### FT-IR analysis

**Figure 4:**
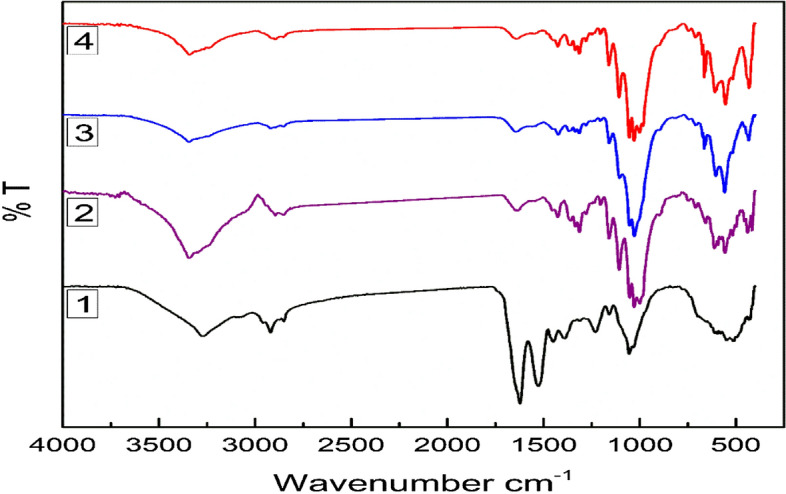
FT-IR spectra of obtained BC membranes using different regimes of cultivation, HS (1), JCP (2), ECPS (3), and EPC (4) media.

FT-IR analysis was applied to examine the structural differences in BC membranes by evaluating the specific functional groups. The results (Fig. 4) show the IR spectra of BC obtained from different regimes of cultivation. The structures of BC membranes from different cultivation regimes are almost similar to one another. The intensity degree of BC obtained from HS media at 1460–1480 cm^−1^, which corresponds to -CH_2_ bending, related to crystallinity and amorphous proportions in cellulosic molecules. BC obtained from HS media shows the highest characteristic peak at 1480 cm^−1^, which refers to the highest BC crystallinity degree, compared with other BC obtained from other cultivation regimes. Notably, other BCs obtained from JCP, ECPS, and ECP media show an amorphous structure with a broad peak at 1465 cm^−1^. On the contrary, the characteristic peaks of BC in the regions at 3347 cm^−1^ of the stretching vibration of -OH groups, at 2927 cm^−1^ of the stretching vibration of C–H groups, and at 1626 cm^−1^ of the deformational vibration of –OH groups of bound water, were similar to those reported by Fan et al.^85^. The bands at 1163 cm^−1^ were attributed to the C1–O–C4 glycosidic link, whereas the bands at 1100, 1060, and 1035 cm^−1^ were assigned to vibrations of C2–O2, C3–O3, and C6–O6, as reported by Kačuráková et al. and Santos et al.^86,87^. Moreover, at 650–850 cm^−1^ the β-1,4 bond vibration was kept stable with the same BC structures.

#### XRD analysis

**Figure 5:**
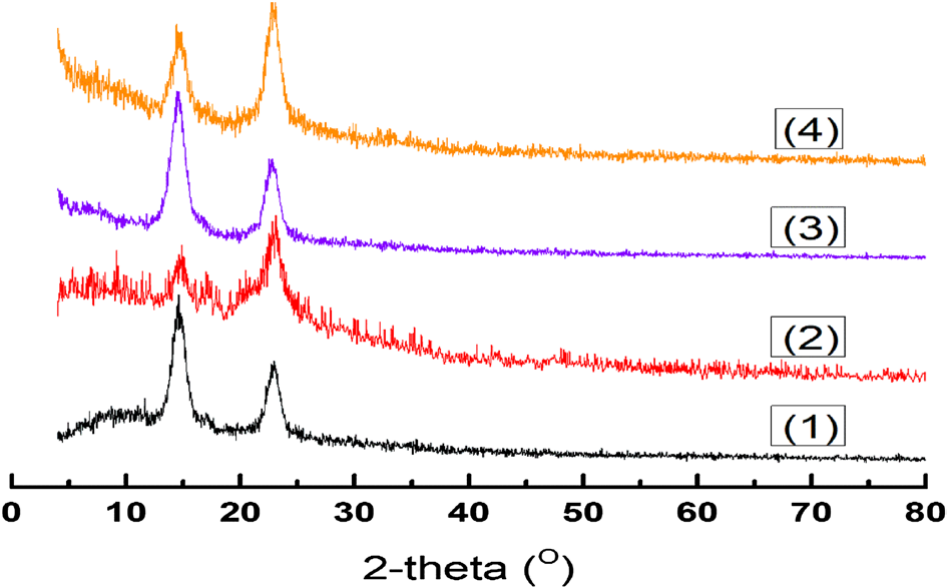
XRD patterns of obtained BC using different regimes of cultivation, HS (1), JCP (2), ECPS (3), and EPC (4) media.

In general, XRD patterns of different cultured BC are similar to patterns of BC obtained from HS media (Fig. 5). Typical XRD patterns obtained from all tested BC membranes demonstrated two characteristic patterns at ~ 2θ 14.6° and 22.8°, which correspond to the typical patterns of cellulose. The crystallographic planes marked as (100) and (110) corresponded to diffraction angles of 14.6° and 22.8°^88–90^. However, these patterns might be resolved and slightly shifted, particularly with BC obtained from JCP media, indicating to the crystallinity/amorphous degree alternation. Therefore, BC obtained from JCP, ECPS and ECP media show less crystallinity (53.33, 87.0 and 69.33%, respectively), than highly crystalline BC obtained from HS media, which reached to 94.44%. Furthermore, XRD analysis indicated that the majority of the BC membranes obtained from JCP, ECPS, and ECP media, were type-1β BC, which is similar to BC obtained from HS media.

#### TGA measurement

**Figure 6:**
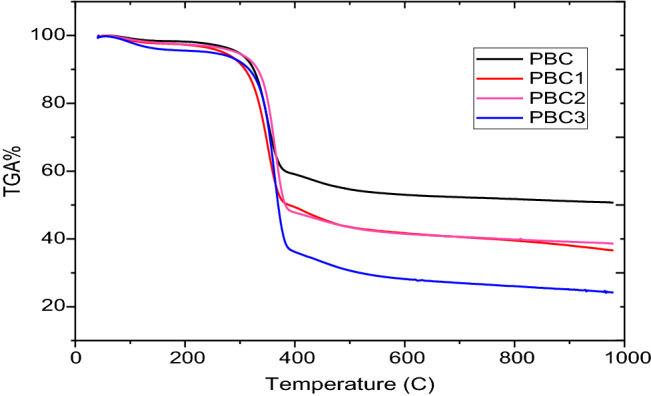
TGA thermograph results of obtained BC using different regimes of cultivation, including HS (PBC), JCP (PBC1), ECPS (PBC2), and EPC (PBC3) media.

Figure 6 shows TGA thermographs of BC obtained from different cultivation regimes. Two significant weight loss stages were observed from ambient to 237 °C as well as from 237 °C to 527 °C. The first significant weight loss may be attributed to water evaporation, whereas the second one at ~ 237 °C corresponded to the degradation of the main cellulose skeleton^91^. All thermographs of BC showed that slight weight loss varied from 5 to 10% during the first stage of thermal decomposition from 25 to 200 °C because of moisture and humidity vaporization and other rapid weight loss at ~ 300 °C resulting from the decomposition of organic matters and cellulosic molecules. T_onset_ temperature (the beginning of the sharp-degradation of second thermal decomposition) varied from 285 to 340 °C, which is assigned to all BC obtained from JCP, ECPS, and ECP media. Notably, the total weight loss was about 65% in the case of BC obtained from HS media, however the total weight loss of BCs obtained from JCP, ECPS, and ECP media was significantly reduced from 65% at 550 °C to 60%, and 55% at 800 °C, respectively. Therefore, BC obtained from JCP, ECPS, and ECP media showed better thermal stability than that obtained from HS media, because of the fibrotic and compacted interior structure of BCs obtained from JCP, ECPS, and ECP media, compared to the regular and less-fibrosis structure of BC obtained from HS media (Fig. 6). These results are consistent with other studies that reported the higher thermal stability of BC obtained from natural sources such as cashew tree residues^92^ and vinasse^80^ than that obtained from standard media.

#### Characterization of green synthesized CuO-NPs

**Figure 7:**
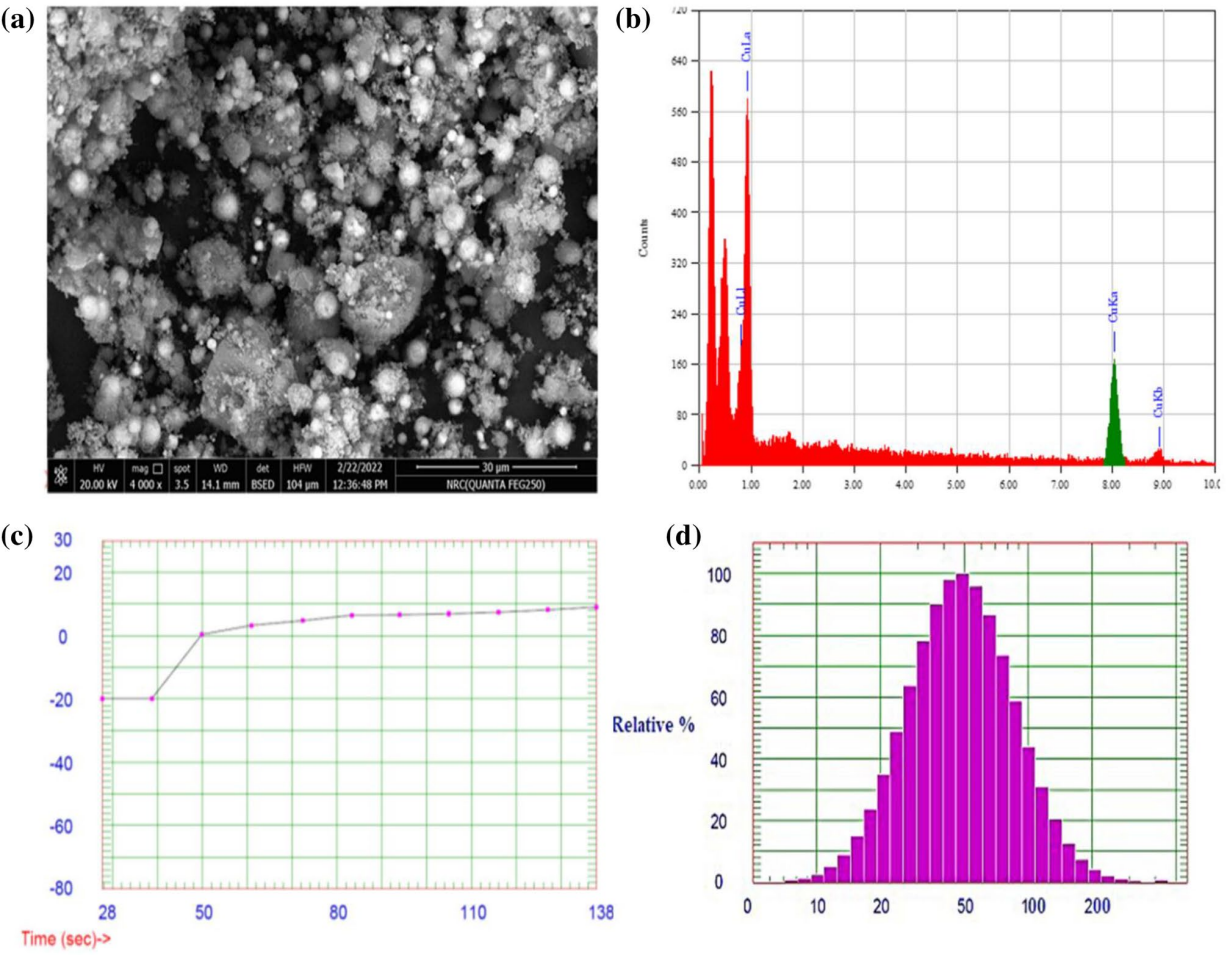
Full characterization of prepared CuO-NPs., (**a**) SEM micrograph (image scale 10 µm, original magnification of 7,000 × and applied voltage of 20 kV), (**b**) EDX analysis, (**c**) zeta potential, and (**d**) particle size analysis.

Metal reduction to NPs could be easily observed through color change in the reaction solution^93^. The surface morphology of synthesized CuO-NPs was investigated using SEM analysis (Fig. 7a). CuO-NPs show good homogeneity, a spherical shape, and appropriate separation. However, few aggregates could be attributed to particle aggregation during washing. EDX spectra show the characteristic absorption peaks of Cu, implying the existence of Cu (Fig. 7b). In addition, particle size distribution analysis shows that the mean particle size diameter of CuO-NPs is approximately 48 nm, whereas the other fraction is about 32.4 nm (Fig. 7d)^94^. Notably, the examined CuO-NPs show a zeta potential value ~ 9.01 mV, which indicates the moderate distribution of nanoparticles, with some particle agglomerations, as further shown in SEM investigation (Fig. 7c).

### Characterization of BC/PVA/Ch/CuO-NPs composite membranes

#### SEM investigation

**Figure 8:**
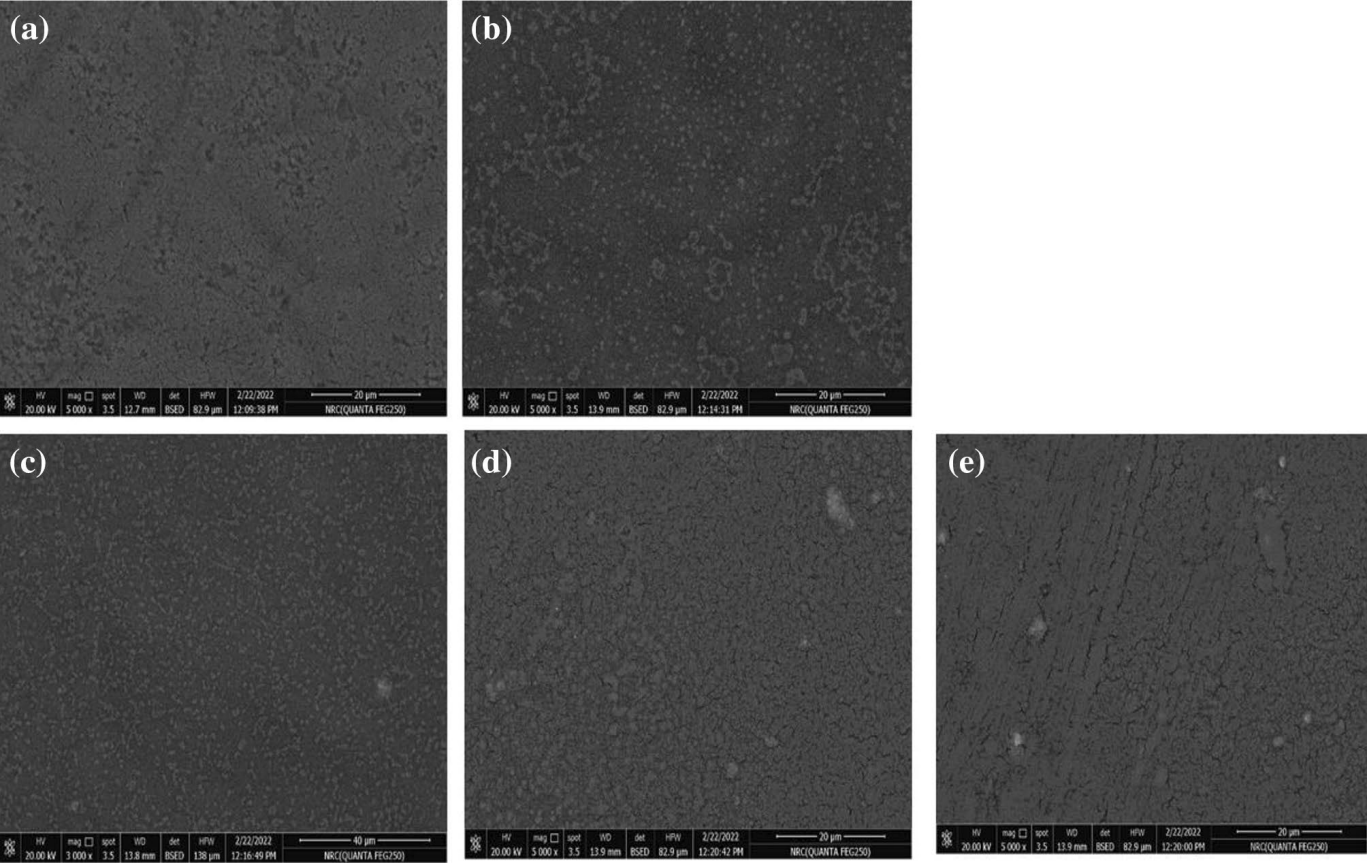
SEM micrographs of BC/PVA/Ch composite membranes loaded with CuO-NPs (0.025, 0.050, 0.075, and 0.100 mg in (**a**, **b**, **c**, **d**, and **e**), respectively). All images were taken with 20μm, original magnification of 5000 x, with applied voltage of 20 kV.

Figure 8 shows the morphological surface structure of BC/PVA/Ch composite membranes loaded with CuO-NPs at different concentrations (0.025, 0.050, 0.075, and 0.100 mg). Based on displayed images, the BC/PVA/Ch membrane shows a uniform, compacted, and less porous surface structure (Fig. 8a), where BC shows a good compatible component of the membrane without any shrinking or failure structure. After the addition of CuO-NPs in different ratios, CuO-NPs were randomly dispersed at the surface, whereas NPs were also exfoliated in a homogenous manner (Fig. 8b–e). An evident aggregation of CuO-NPs was observed when NPs were incorporated at the highest concentration (i.e. 0.100 mg, Fig. 8e). The aggregated NP_S_ might cause the formation of a few cracks and a rigid surface membrane structure.

#### FT-IR analysis

**Figure 9:**
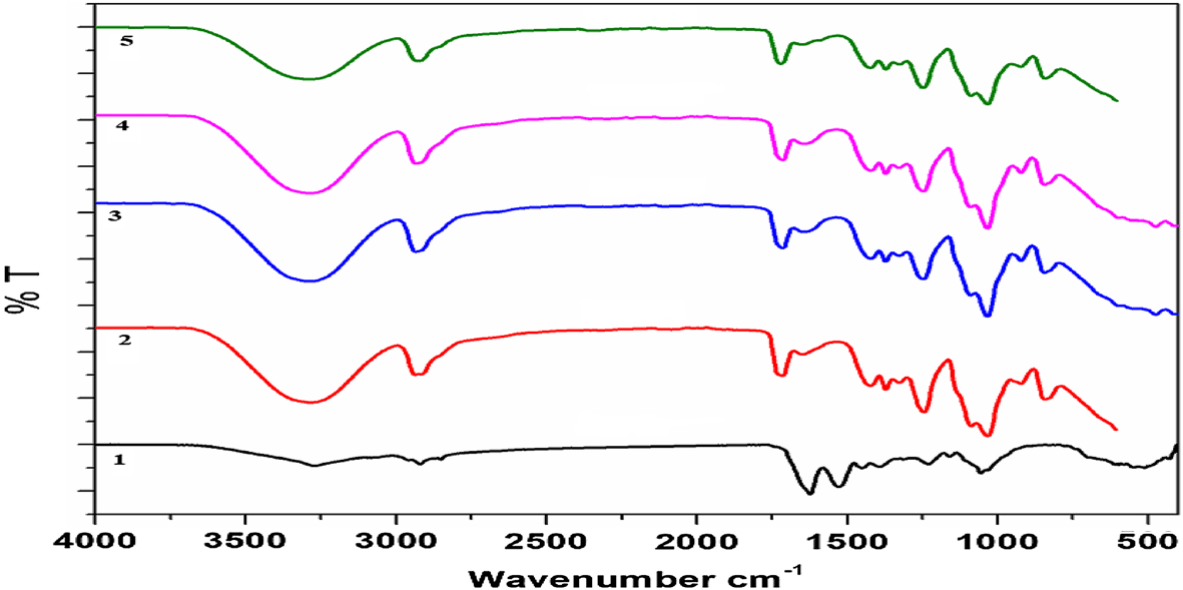
FT-IR analysis of BC/PVA/Ch (1), BC/PVA/Ch/CuO-NPs1 (2), BC/PVA/Ch/CuO-NPs2 (3), BC/PVA/Ch/CuO-NPs3 (4), and BC/PVA/Ch/CuO-NPs4 (5) composite membranes.

The interaction among BC, PVA, Ch, and CuO-NPs was analyzed by FT-IR analysis. Figure 9 shows the FT-IR spectra of BC/PVA/Ch/CuO-NPs compared with BC/PVA/Ch as a composite control. The FT-IR spectrum of CuO-NPs depicts the distinctive peaks at 3290 cm^−1^ and 1250 cm^−1^ for BC/PVA/Ch/CuO-NPs membranes with different ratios of CuO-NPs. This finding indicated that CuO-NPs were adsorbed in the fiber network through physical bonding, and the chemical structure of fibers was not altered by the adsorption of CuO-NPs.

#### XRD analysis

**Figure 10:**
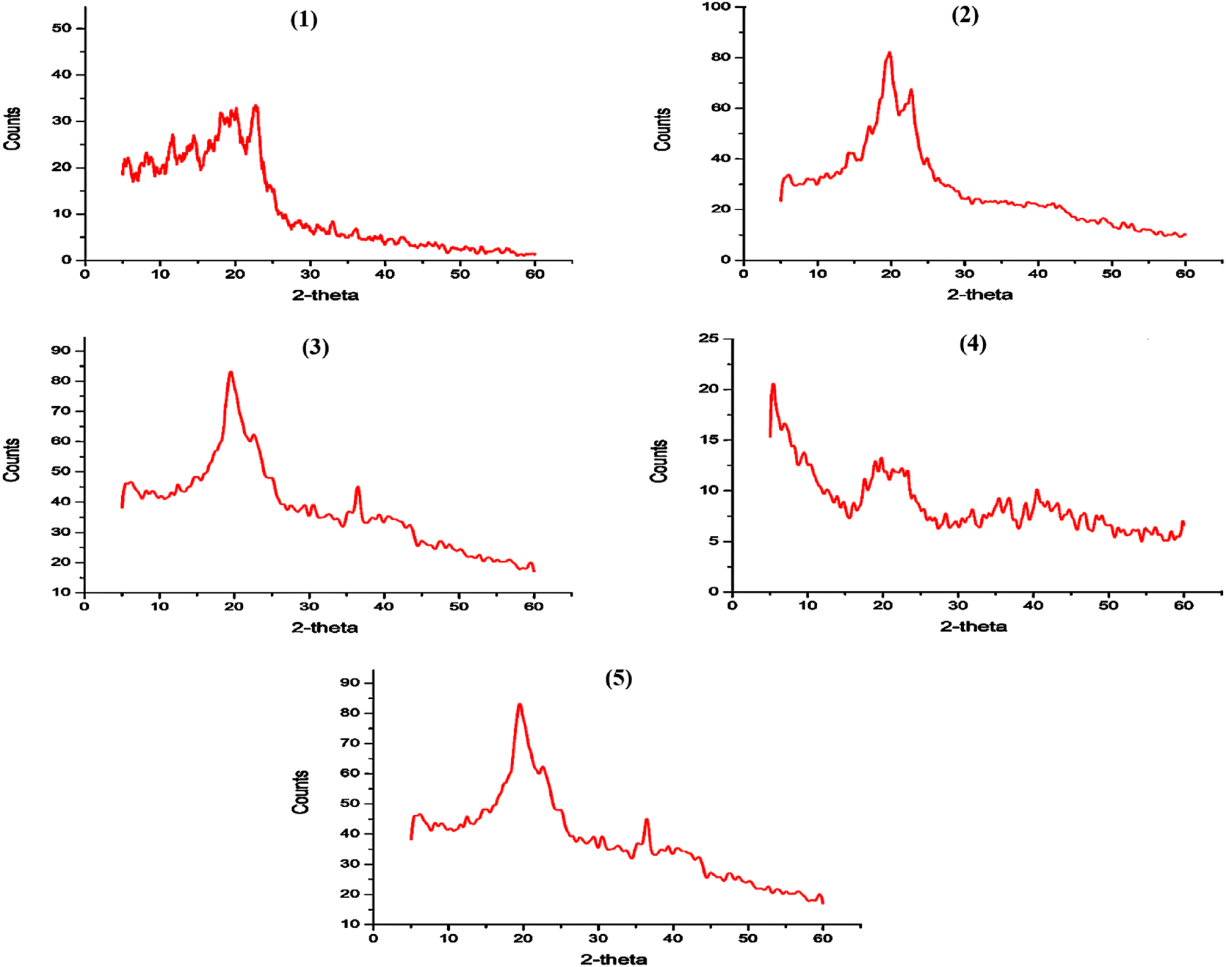
XRD patterns of BC/PVA/Ch (1), BC/PVA/Ch/CuO-NPs1 (2), BC/PVA/Ch/CuO-NPs2 (3), BC/PVA/Ch/CuO-NPs3 (4), and BC/PVA/Ch/CuO-NPs4 (5) composite membranes.

XRD patterns of cross-linked BC/PVA/Ch composite membranes were loaded with different concentrations of CuO-NPs (0.025, 0.050, 0.075, and 0.100 mg Fig. 10). The displayed patterns showed that the amorphous nature of these materials is increasing in the following order: BC/PVA/Ch > BC/PVA/Ch loaded CuO-NPs. However, the crystallinity structures of membranes are increasing and they vary significantly (from 65 to 100%) with the increase of CuO-NPs content incorporation at amorphous diffraction peaks of 2θ = 20°, which is easily indicated by the BC/PVA/Ch composite membrane. Furthermore, two featured patterns of CuO-NPs at 2θ = 35° and 40^o^ are detected with the highest incorporated CuO-NPs (*i.e.* 0.075 and 0.100 mg) in BC/PVA/Ch membranes. In addition, the crystalline regions, which refer to the presence of BC in membranes, are found at two positions, 2θ = 14° and 22°. The same peaks are found for other samples, which differ only in crystallinity percentage because of the incorporation of CuO-NPs. The current findings are consistent with the results obtained by Jozala et al. and Abdeen et al.^95,96^.

#### Physicochemical characterization of BC/PVA/Ch/CuO-NPs composite membranes

**Figure 11:**
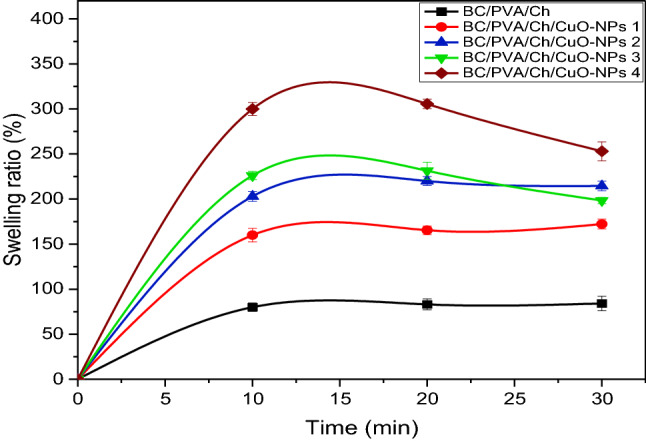
The swelling ratio (SR%) of the prepared BC/PVA/Ch at different concentrations of CuO-NPs.

The thickness, moisture content (MC), and composite content (CC) of BC/PVA/Ch and BC/PVA/Ch/CuO-NPs composite membranes are shown in Table 2. The thickness, among the most important membrane characteristics, was directly reflected in the membrane applicability in different fields^97^, hence, thickness of the prepared composite membranes was measured after drying using an electronic digital micrometer. The results (Table 2) indicated a significant gradual enhancement in the membranes thickness proportional to CuO concentration. The maximum membrane thickness was detected in BC/PVA/Ch/CuO-NPs4 (0.31 ± 0.043 mm) representing a 2.4-fold increase compared to the BC/PVA/Ch control membrane (0.13 ± 0.022 mm). The enhancement in BC/PVA/Ch/CuO-NPs thickness with the CuO-NPs concentration increase could be attributed to the uniform distribution of CuO-NPs throughout the membrane matrix^98,99^. The MC values of the prepared membranes ranged from 16.2 to 22.5%. The neat BC/PVA/Ch membrane showed higher MC than PVA/Ch/CuO-NPs composite membranes. It was observed that the MC of BC/PVA/Ch was decreased with increasing the CuO-NPs concentrations. The strong interaction of CuO-NPs with BC/PVA/Ch chains lowers the availability of OH groups, resulting in a reduction in hydrophilicity and MC value^100,101^. The CC values indicate the cross-linked of polymer chains in the composite structure. The crystallinity and the degree of cross-linking depend on the interaction between the components of the composite. Table 2 represents the varied CC values of tested composite membranes between 15.3 and 28.8%. It was observed that, with increasing the amount of CuO-NPs in the composite structure, the CC would diminish, these results are fully consistent with other reports^102,103^. Water uptake is regarded as a vital parameter for biomaterial applications. The swelling ratio (SR%) for all composite membranes increased progressively over time to the equilibrium swelling-state as indicated in Fig. 11. Generally, the increase in CuO-NPs concentration in composites brought a higher SR% and reduced the time required for equilibrium swelling-state. The maximum SR was in BC/PVA/Ch/CuO-NPs4 (313.6 ± 5.21%) after 20 min representing a 3.6-fold increase compared to the BC/PVA/Ch control membrane (87.3 ± 5.5% after 80 min). BC/PVA/Ch/CuO-NPs2 and BC/PVA/Ch/CuO-NPs3 require the same time (20 min) for equilibrium swelling-state with SR of 2204.98 and 231.49.55%, respectively. The results are comparable to BC/PVA/Ch/CuO-NPs1 and BC/PVA/Ch required 30 and 80 min, respectively, for attending SR of 182.2 ± 5.5 and 87.3 ± 5.5%. The higher CuO-NPs concentration enhanced the penetration of water molecules to balance the osmotic pressure differences between membranes and surrounding media, which increases the SR%^104^. The obtained results are consistent with a previous study that reported the enhancement in the SR% of oxidized starch/PVA hydrogels by CuO-NPs incorporation^104^, however, other NPs like ZnO, Au-, and Fe_3_O_4_ had no effect on the SR when combined with other composites^105–107^.

**Table 2:** Physicochemical characterization of BC/PVA/Ch/CuO-NPs composite membranes including: thickness, moisture content (MC) and composite content (CC) of BC/PVA/Ch at different CuO-NPs concentrations.

Composite membranes	Thickens(mm)	MC (%)	CC (%)
BC/PVA/Ch	0.13 ± 0.022^c^	22.5 ± 2.273^a^	28.8 ± 1.96^a^
BC/PVA/Ch/CuO-NPs1	0.22 ± 0.016^b^	19.3 ± 2.005^b^	20.6 ± 2.417^b^
BC/PVA/Ch/CuO-NPs2	0.25 ± 0.016^b^	18 ± 0.817^b^	16.5 ± 0.707^c^
BC/PVA/Ch/CuO-NPs3	0.27 ± 0.008^ab^	17.4 ± 0.7118^b^	15.5 ± 1.715^c^
BC/PVA/Ch/CuO-NPs4	0.31 ± 0.043^a^	16.2 ± 1.115^b^	15.3 ± 1.225^c^

All values were expressed as Mean ± standard deviation. Different letters are significantly different in ascending order where a > b > c > d > e within the same column at p < 0.05.

### Biological evaluation of BC/PVA/Ch/CuO-NPs composites

#### Antimicrobial activity

**Figure 12:**
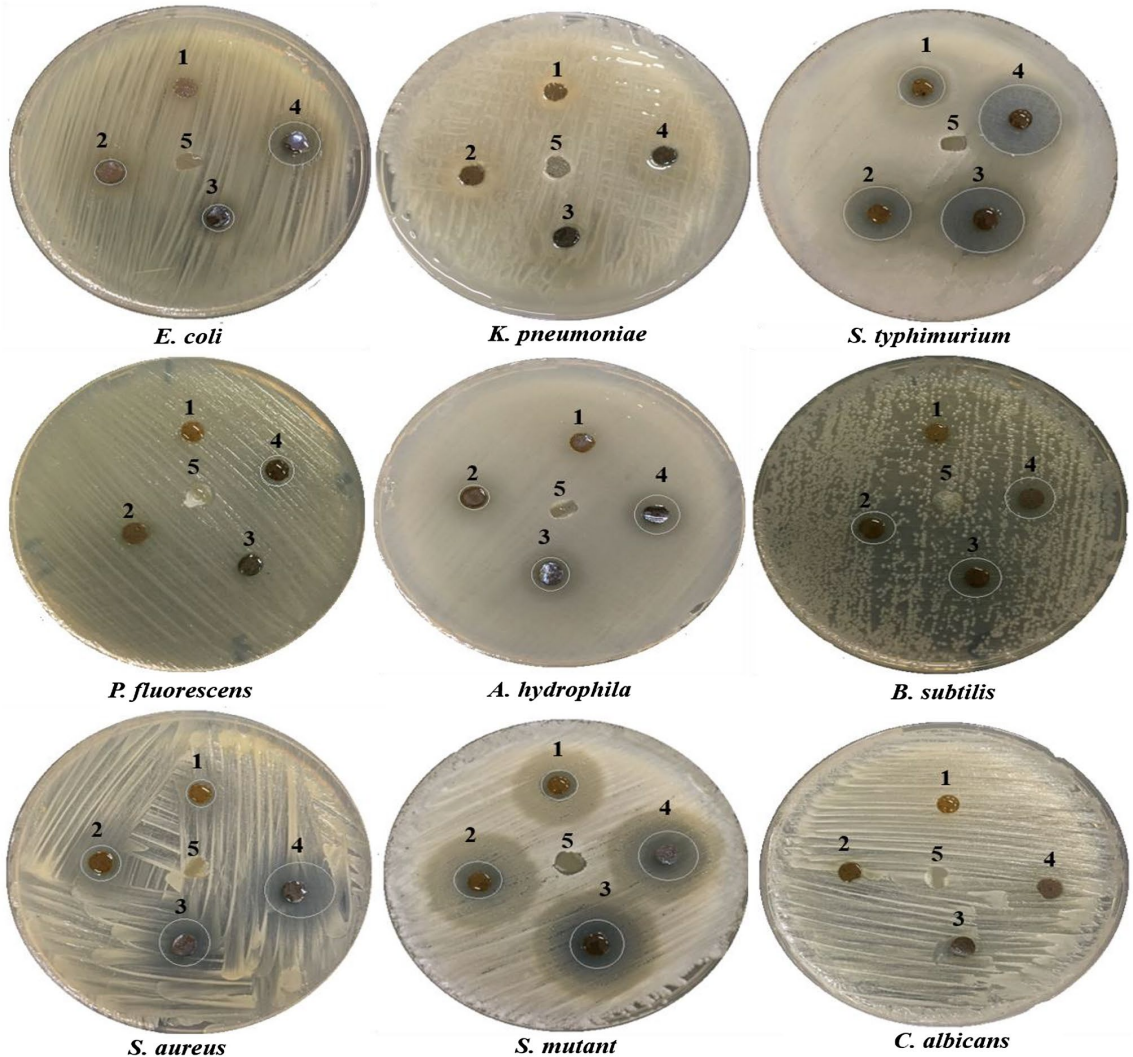
Disk diffusion method for antimicrobial activity expressed as halo-zones of the prepared BC/PVA/Ch at four CuO-NPs concentrations including: 1 (0.025), 2 (0.050), 3 (0.075), and 4 (0.100) compared with 5 (CuO-NPs/free BC/PVA/Ch) against nine pathogenic microorganisms.

Disk diffusion method was applied to evaluate the antimicrobial activity of the prepared BC/PVA/Ch composite at different CuO-NPs concentrations. The results (Table 3 and Fig. 12) indicated significant effects of the prepared BC/PVA/Ch/CuO-NPs composites against seven of the tested pathogenic microbes, compared with the BC/PVA/Ch membranes that showed no antimicrobial activity against the nine applied pathogens. The antimicrobial activity increased with the increase of CuO-NPs concentration in all tested susceptible microbes, indicating the role of loaded CuO-NPs in the detected activity. Based on the inhibition zone diameter (Table 3), the highest antibacterial activity against Gram-positive was reported by BC/PVA/Ch/CuO-NPs4 against *S. aureus* (26 ± 2.63 mm) followed by *S. mutans* (17 ± 0.76 mm). The maximum antibacterial activity against Gram-negative was toward *S. typhimurium* (22 ± 3.4 mm) followed by *E. coli* (15 ± 1.78 mm), where the lowest activity was against *P. fluorescens* (9 ± 1.78 mm). This varied antibacterial activity, based on the pathogen type, could be attributed to the variation in the outer envelope structure among different bacterial genera^108^. On the contrary, no antimicrobial activity was detected toward *K. pneumoniae and C. albicans* at the four applied CuO-NPs concentrations. The resistance of *K. pneumoniae* to the prepared composites could be attributed to the surrounding capsule polysaccharides that averts the accessibility of Cu ions to cells. In addition, the lack of antifungal activity against *C. albicans* indicates that the prepared composites specifically target the prokaryotic membrane structures. Similar results were obtained from CuO-NPs synthesized from bioinspired sources, which exhibit excellent antimicrobial activity against different bacterial and fungal pathogens^109^. Several studies investigated the antimicrobial activity of composites containing CuO-NPs as Ch capping of CuO-NPs^48^, BC/CuO-NPs^49,110^, BC/graphene oxide/CuO-NPs^50^, and BC/ZnO/CuO-NPs^111^. The antimicrobial activity of CuO-NPs has been widely reported, however, its antimicrobial mechanism is remains unclear. The binding of CuO-NPs to the lipid layer of the bacterial cell membrane, which interferes with membrane permeability and nutrient uptake, was proposed to elaborate such activity^112^. Furthermore, Cu ions from CuO-NPs could inactivate essential cellular peptides that interferes with DNA replication and ATP production^113^. Finally, the role of Cu ions in generating reactive oxygen species (ROS) was also reported, resulting in adverse cellular oxidation and cell death^114^.

**Table 3:** The antimicrobial activity of the prepared BC/PVA/Ch/CuO-NPs composites at four CuO-NPs concentrations compared to BC/PVA/Ch (negative control) against nine pathogenic microorganisms.

Organisms	Diameters of inhibition zone (mm)				
	BC/PVA/Ch	BC/PVA/Ch/CuO-NPs			
		1	2	3	4
*E. coli*	0.0 ± 0.0	0.0 ± 0.0	7 ± 0.98	9 ± 1.45	15 ± 1.78
*K. pneumoniae*	0.0 ± 0.0	0.0 ± 0.0	0.0 ± 0.0	0.0 ± 0.0	0.0 ± 0.0
*S. typhimurium*	0.0 ± 0.0	14 ± 1.02	16 ± 1.98	20 ± 2.16	22 ± 3.4
*P. fluorescens*	0.0 ± 0.0	0.0 ± 0.0	0.0 ± 0.0	0.0 ± 0.0	9 ± 1.78
*A. hydrophila*	0.0 ± 0.0	0.0 ± 0.0	7 ± 1.11	8 ± 0.78	10 ± 1.47
*B. subtilis*	0.0 ± 0.0	0.0 ± 0.0	11 ± 0.79	13 ± 1.23	15 ± 2.24
*S. aureus*	0.0 ± 0.0	21 ± 2.09	23 ± 2.25	25 ± 2.47	26 ± 2.63
*S. mutant*	0.0 ± 0.0	9 ± 1.45	12 ± 2.3	16 ± 1.78	17 ± 0.76
*C. albicans*	0.0 ± 0.0	0.0 ± 0.0	0.0 ± 0.0	0.0 ± 0.0	0.0 ± 0.0

All values were expressed as Mean ± standard deviation.

#### In vitro cytotoxicity and anticancer evaluation of the prepared composites

**Figure 13:**
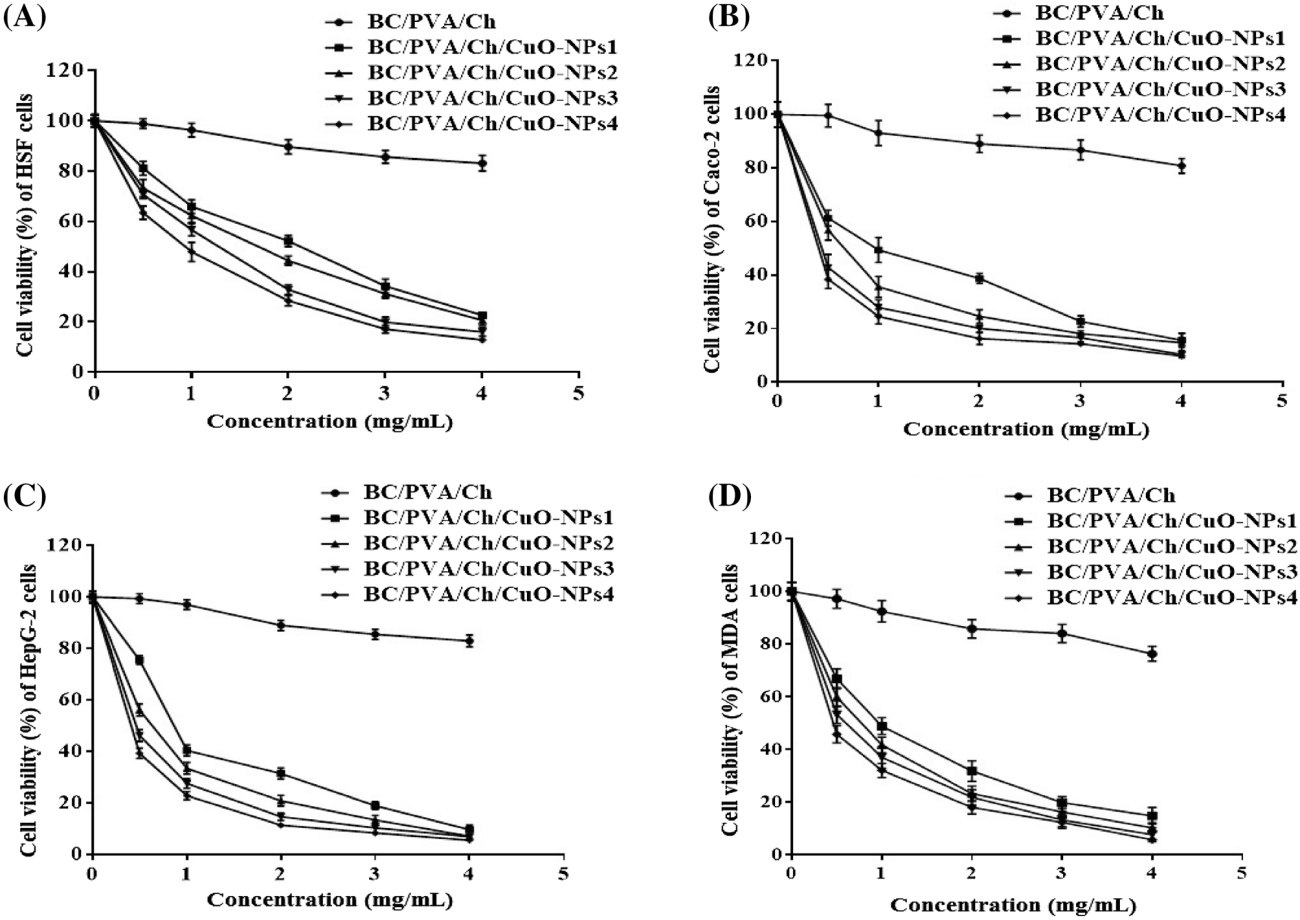
Effect of BC/PVA/Ch at four CuO-NPs concentrations on the cell viability of both normal and cancer cells. Normal HSF cells (**A**) and cancer cell lines of Caco-2 (**B**), HepG-2 (**C**), and MDA (**D**) cells were incubated with discs of the prepared composites at various weights of 0–4.0 mg/mL for 48 h and the viability of all tested cells was assayed by MTT method. All values are representing the average values from three experimental trials and expressed as mean ± SEM.

Cancer is one of the leading causes of death worldwide, with about 70% mortality rate that has increased in low-to-middle income countries. In 2020, about 19.3 million new cancer cases with almost 10.0 million deaths were estimated worldwide^115^. The serious side effects, coupled with the lower efficiency of most currently applied chemotherapy, force the necessity for safer and more efficient treatments. The use of nanotechnology may provide a rational alternative for producing a remedial drug. The potential activity of some nanometals, such as CuO-NPs, has been given a potent role as tumor inhibitors owing to their low cytotoxicity and unique features. Though Cu is a multifunctional element that supports various cellular processes, exposure to high Cu doses is associated with several health complications^116^. Herein, a rapid colorimetric assay of MTT was used to evaluate the cytotoxicity of the prepared BC/PVA/Ch/CuO-NPs in both normal and cancer cell lines. The viability of normal human HSF cells was determined to be 2.19–5.03-fold higher than cancer cells after treatment with the prepared BC/PVA/Ch/CuO-NPs (Table 3). Our results indicate that IC_50_ values of the prepared composite membranes were decreased by increasing the concentration of CuO-NPs in the tested membrane. Both Table 4 and Fig. 13 emphasize the improvement in the anticancer effect of the prepared composite membranes in a dose-dependent manner on all tested cells. Furthermore, Fig. 13 shows a highly significant increase in the safety of using the prepared composite membranes toward normal HSF cells with high selectivity against all tested cancer cell lines. However, the control membrane (BC/PVA/Ch) had not shown any cytotoxicity against both normal and cancer cells with greater IC_50_ values. The results indicated that Caco-2 cells are the most susceptible cells to treatment with the prepared composite membranes, revealing IC_50_ values of 0.99, 0.58, 0.33, and 0.27 (mg/mL) with SI values of 3.92, 3.72, 5.03, and 4.0 for BC/PVA/Ch/CuO-NPs1, BC/PVA/Ch/CuO-NPs2, BC/PVA/Ch/CuO-NPs3, and BC/PVA/Ch/CuO-NPs4, respectively.

**Table 4:** IC_50_ (mg/mL) and SI values of the prepared BC/PVA/Ch at four CuO-NPs concentrations (0.025, 0.050, 0.075, and 0.100 mg, respectively) against HSF, Caco-2, HepG-2, and MDA cell lines after treatment for 48 h.

Composite membranes	HSF	Caco-2		HepG-2		MDA	
	IC_50_	IC_50_	SI	IC_50_	SI	IC_50_	SI
BC/PVA/Ch	13.2 ± 0.48^a^	11.89 ± 1.02^a^	1.11 ± 0.04^a^	12.99 ± 0.59^a^	1.02 ± 0.03^a^	12.46 ± 1.7^a^	1.05 ± 0.04^a^
BC/PVA/Ch/CuO-NPs1	3.88 ± 0.14^b^	0.99 ± 0.0 ^b^	3.92 ± 0.14^b^	1.12 ± 0.15^b^	3.46 ± 0.13^a^	1.17 ± 0.23^b^	2.19 ± 0.12^a^
BC/PVA/Ch/CuO-NPs2	2.16 ± 0.15^c^	0.58 ± 0.01^b^	3.72 ± 0.25^b^	0.68 ± 0.06^b^	3.18 ± 0.22^a^	0.85 ± 0.14^b^	2.54 ± 0.18^a^
BC/PVA/Ch/CuO-NPs3	1.66 ± 0.13^d^	0.33 ± 0.009^b^	5.03 ± 0.39^b^	0.45 ± 0.03^c^	3.69 ± 0.29^a^	0.67 ± 0.16^b^	2.48 ± 0.19^a^
BC/PVA/Ch/CuO-NPs4	1.08 ± 0.11^e^	0.27 ± 0.008^b^	4.0 ± 0.41^c^	0.33 ± 0.01^c^	3.27 ± 0.33^b^	0.48 ± 0.05^b^	2.25 ± 0.23^b^

All IC_50_ values were expressed as mean ± standard deviation. Different letters are significantly different in ascending order where a > b > c > d > e within the same column at p < 0.05.

**Figure 14:**
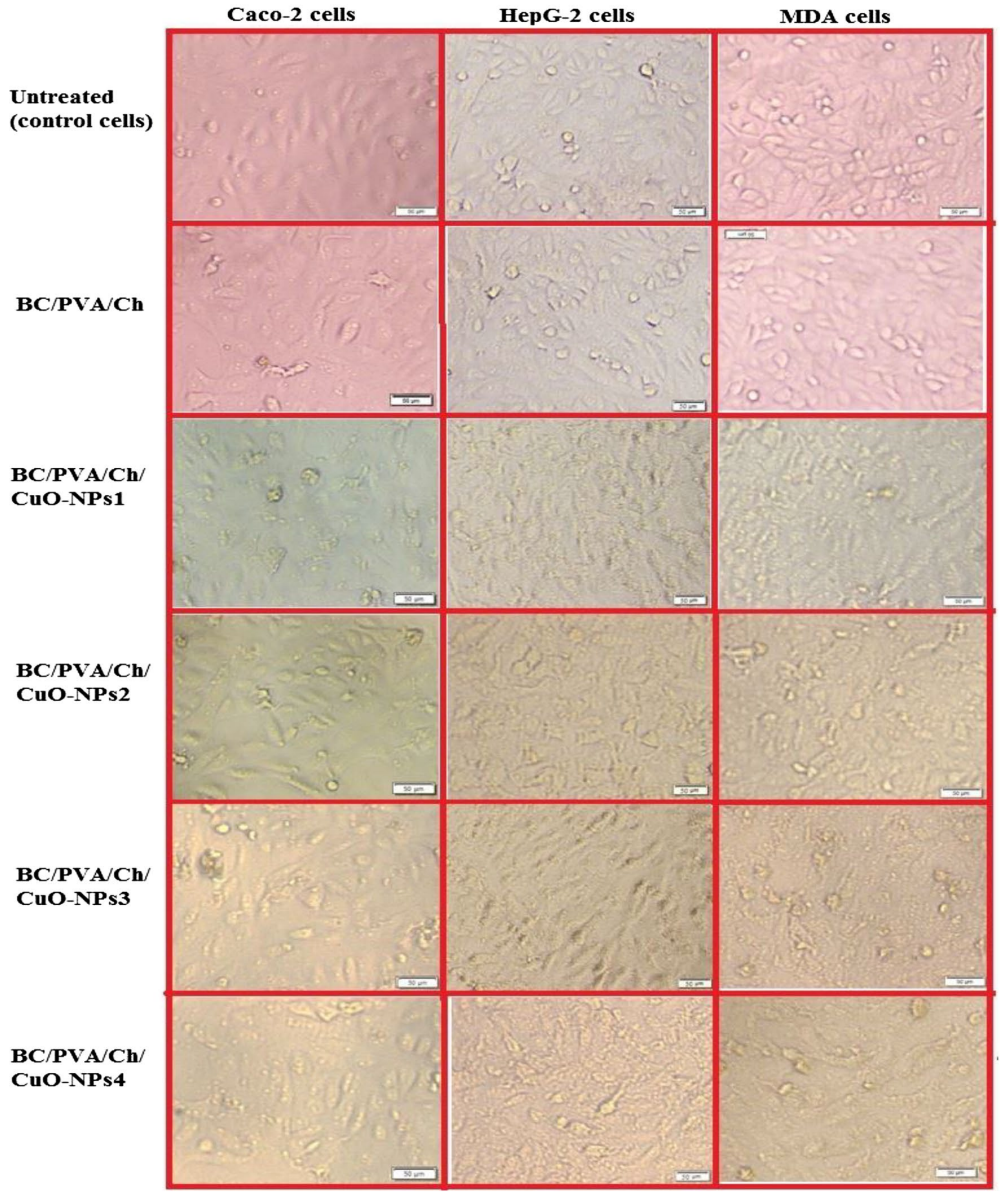
Effect of BC/PVA/Ch at four concentrations of CuO-NPs on the morphological changes of Caco-2, HepG-2, and MDA cancer cell lines as shown under an inverted phase-contrast microscope. Cells were treated at the IC_50_ value of each prepared composite membrane and untreated (control) cells were included as negative references.

The proportional morphological investigation of the three cancer cell lines upon treatment with IC_50_ values of the prepared composite membranes was presented in Fig. 14 as compared to untreated cells (negative references). The live-mode microphotographs indicated that the morphology of all the studied cancer cells was extremely modified after 48 h of treatment with the prepared composite membranes. The morphological modifications include noticeable nuclear condensation, cell shrinkage, and blabbing in all treated cancer cells (Fig. 14), with no detectable changes in non-treated cells (negative controls). Based on these findings, it appears that the prepared composite membranes provoke the apoptosis pathway to trigger their anticancer effect. The prepared membranes might disrupt cellular membranes and generate vacuoles in the treated cancer cells. Thus, the CuO-NPs and chitosan-based membranes could modify the metabolism of cancer cells and enhance apoptosis, which finally leads to cancer cell death. In addition to alteration in the cellular and nuclear morphology, the ROS production through Cu ions (especially hydroxyl radical) could force DNA damage in the cancer cells, which enhances the cell cycle arrest mediating cell death^117–120^. Our findings confirmed the high selectivity and synergistic anticancer activity of BC composites containing CuO-NPs and chitosan against cancer cells, which is in line with many recent studies^53,121–123^. Collectively, the anticancer results support the use of the newly prepared composite as potential anticancer candidates in the treatment of various cancer types while remaining safe to surrounding normal cells.

#### Larvicidal activity

Mosquitoes frequently participate in the spread of several diseases such as malaria, filariasis, dengue fever, chikungunya, and Japanese encephalitis. At present, mosquito-borne diseases are increasing worldwide, indicating major health challenges^124,125^. The results of larval mortality tests (Table 5) revealed that BC/PVA/Ch/CuO-NPs exhibited lethal effects against all stages of larval and pupal instar through their contact areas compared with control (ddH_2_O) that exhibited no mortality. The percentage mortality results of BC/PVA/Ch/CuO-NPs against larval and pupal instar stages were positively dose-dependent, which increased with concentration. In research, the high mortality (%) recorded at BC/PVA/Ch/CuO-NPs4 (0.100 mg) during the immature stage of *Aedes aegypti* (*A. aegypti*) was 93.0, 81.0, 72.9, and 65.0% for I, II, III, and IV larval instars, respectively, whereas mortality percentage against pupal stage exhibits only 55.0%. The minimum concentration of BC/PVA/Ch/CuO-NPs1 (0.025 mg) recorded a mortality percentage of 12.9% for the immature pupal stage and 32.1, 28.4, 22.0, and 19.2% for I, II, III, and IV larval instars, respectively. The results are consistent with those of Salem et al., who found an increase in the larvicidal activity with the increase of Se-NPs concentration and reported 100% larvae mortality at 100 ppm of Se-NPs as compared with other concentrations, which attained 90.6%, 70.3%, 50.3%, and 43.3% larval mortality by treating with 75.0, 50.0, 25.0, and 20.0 ppm Se-NPs, respectively^126^. Based on the observed results shown in Table 5, the larvicidal and pupicidal activities were influenced by an increase in CuO-NPs concentrations in the fabricated membranes. In general, instar I is more sensitive to treatment by the fabricated membranes than other instars. For pupal instar, the pupicidal activity ranged from 12.90% to 55%. Alsharif et al., showed that LC_50_ for Ag-NPs synthesized by strains A1-5, H1-1, and A6-2 on the 3rd larval instar of *A. aegypti* was 12.5, 12.8, and 12.7 ppm, respectively, against mosquito vector^127^. The efficacy of NPs provided mortality percentages of pupa because of treatment with the highest MgO-NPs concentration (25 ppm) accounting for 69.2–2.8%, with LC_50_ of 16.5 ppm and LC_90_ of 29.8 ppm, as reported by Fouda et al.^128^. Hassan et al. have reported the efficacy of nanoparticles against mosquitoes where the LC_50_ (concentration of MgO-NPs that inhibit 50% of the population) and LC_90_ (concentration of MgO-NPs that inhibit 90% of the population) were 2.21 µg/mL and 10.71 µg/mL, respectively^129^. Another study has addressed the larvicidal efficacy of the aqueous and green synthesized Ag-NP with the 3rd larval instar of *Anopheles subpictus* (LC_50_ values of 11.82 and 0.69 ppm) and *Culex quinquefasciatus* (LC_50_ of 13.65 and 1.10 ppm)^130^. Collectively, the results of this study indicated that green synthesized CuO-NPs have good larvicidal activity toward *A. aegypti* which is attributed to their high penetration capacity. This high penetration capacity could be elucidated by the high surface-to-volume ratio of CuO-NPs, resulting in the disruption of organelles and enzymes in young juvenile instars, which are more susceptible to CuO-NPs action than higher instars. This study is the first to investigate the larvicidal and pupicidal activities of BC/PVA/Ch with different concentrations of green synthesized CuO-NPs against *A. aegypti*. Further research on CuO-NPs could lead to a potential vector control avenue.

**Table 5:** Larvicidal and Pupicidal activity of BC/PVA/Ch composite with four different concentrations of CuO-NPs against *A. aegypti* mosquito.

Composite membranes	Targeted instars (Mortality %)				
	I	II	III	IV	Pupa
BC/PVA/Ch	0.0 ± 0.0^e^	0.0 ± 0.0^e^	0.0 ± 0.0^e^	0.0 ± 0.0^e^	0.0 ± 0.0^e^
BC/PVA/Ch/CuO-NPs1	32.10 ± 1.92^d^	28.40 ± 2.74^d^	22.00 ± 2.01^d^	19.20 ± 2.16^d^	12.90 ± 2.38^d^
BC/PVA/Ch/CuO-NPs2	58.00 ± 1.70^c^	53.80 ± 2.28^c^	42.60 ± 2.64^c^	37.40 ± 2.30^c^	25.20 ± 3.39^c^
BC/PVA/Ch/CuO-NPs3	71.00 ± 1.58^b^	66.00 ± 2.12^b^	56.20 ± 1.54^b^	44.20 ± 3.11^b^	39.40 ± 2.96^b^
BC/PVA/Ch/CuO-NPs4	93.00 ± 3.16^a^	81.00 ± 2.44^a^	72.90 ± 2.23^a^	65.00 ± 2.14^a^	55.00 ± 2.91^a^

All values were expressed as Mean ± standard deviation. Different letters indicate significant difference, which are arranged in ascending order within the same column at *P* < 0.05: a > b > c > d > e.

## Conclusion

The results of this study demonstrated the significant utilization of enzymatically hydrolyzed cantaloupe (ECP) to promote BC production by using the *L. plantarum* AS.6 strain. The production of BC was enhanced about 1.6-fold when compared with the standard HS media, with an estimated weight of 3.49 g/L. The produced BC was fabricated with Ch and PVA and reinforced with green synthesized CuO-NPs at different concentrations to provide potent synergistic activities against a wide range of multi-resistant bacteria and cancer types to normal cells in a safe manner. The novel fabricated composite exhibited potent antibacterial activity against Gram-positive and Gram-negative bacteria, including *S. aureus*, *S. mutans*, *S. typhimurium*, *E. coli*, and *P. fluorescens*. The findings of the anticancer activity revealed the potent effect of the novel fabricated composite against different cancer cell lines, including colon (Caco-2), liver (HepG-2), and breast (MDA) cells, with significant degrees of selectivity to cancer cells in a dose-dependent manner. This anticancer selectivity could be mediated through apoptosis induction in treated tumor cells. The novel fabricated composite showed potent lethal effects against all stages of *A. aegypti* larval and pupal instars through their contact areas as compared with the control. These results promote the use of a novel fabricated composite as a drug carrier system with significant antibacterial, anticancer, and larvicidal activities, which could pave the way for other studies on drug delivery systems based on the newly prepared composites.

## Materials and methods

### Materials

Pomegranate (*Punica granatum*) and cantaloupe (*Cucumis melon L.*) fruits were purchased from a local market (Egypt). Copper (II) nitrate trihydrate (Cu(NO_3_)_2_·3H_2_O, obtained from Aladdin Co. Ltd. (Shanghai, China) was used as a copper source. Glucose, yeast extract, and sodium hydroxide were obtained from Fisher Scientific (United Kingdom). Peptone was obtained from Biolife (Italia). MgSO_4_, and KH_2_PO_4_ were obtained from Riedel–dehaen (Germany). Acetic acid and disodium phosphate were obtained from Winlab (Italia). Ethanol 99.8% was obtained from Oxoid Ltd. (United Kingdom). All other chemicals were of analytical grade and were used without any further purification. Double-distilled water (ddH_2_O) was used for all experiments.

### Methods

#### Enzymatic hydrolysis of CP

The enzymatic hydrolysis of CP was performed as described by Saleh et al.^131^ with slight modifications. Firstly, the CP juice was performed as: a fresh CP (20 g) were sliced into small pieces and dispersed in 100 mL of acetate buffer (0.1 M, pH 4.7), then minced by a high-speed blender for 5 min. Enzymatic hydrolysis was performed in a 250 mL flask containing 50 mL of CP juice with 1 mL of cellulases (*Trichoderma reesei* ATCC 26,921 cellulases 89.4 FPU/mL; Sigma, Aldrich, USA). The flask was incubated at 50 °C for 5 days at 75 rpm. At intervals, samples were drawn and centrifuged for 10 min at 11,000 rpm. The CP hydrolysis was monitored by measuring the total carbohydrate, glucose, and reducing sugars liberated during the hydrolysis course. Total carbohydrates were evaluated by the anthrone-sulfuric acid method according to Ludwig et al.^132^, glucose concentration was determined using a glucose assay kit (Oxidase), and reducing sugars released were determined with the dinitrosalicylic acid (DNS) method^133^. The enzymatic hydrolysis rate of CP was calculated^134^ according to the following equation:

1 $$ {\text{Hydrolysis}}\;{\text{rate}} = \frac{{{\text{Glucose}}\;{\text{concentration}}\left( {{\text{g}}/{\text{L}}} \right)}}{{{\text{Time}}\left( {\text{h}} \right)}} \times 100 $$

#### Strain and preinoculum preparation

*Lactiplantibacillus plantarum* AS.6 (*L. plantarum* AS.6) was isolated and screened from rotten apple as reported in our previous work by Saleh et al.^6^. The BC production isolate (*L. plantarum* AS.6) was maintained on HS agar slants, transferred and stored at 4 °C until used. A pure single colony of *L. plantarum* AS.6 was cultured in HS media containing (g/L) glucose 20, yeast extract 5, peptone 5, disodium hydrogen phosphate 2.7, citric acid 1.15, and ethanol 5 ml/L at pH 5.5 and incubated at 200 rpm using a shaker incubator at 30 °C for 48 h^135^.

#### BC production and purification

Four different media preparations were used for BC production as shown in Table 6: HS media was used as standard media, fresh juice was used as CP media, and enzymatic hydrolysis was performed on CP juice media with and without supplementation with the components of HS media except for glucose. The obtained media were labeled as HS, JCP, ECP, and ECPS. The final pH was adjusted to 5.5 before sterilization at 120 °C for 15 min. All prepared media were inoculated with 10% *L. plantarum* AS.6 and incubated at 30 °C for 7 days under static conditions. At the air–liquid interface, the generated BC membrane was collected and washed several times with ddH_2_O to remove medium residues. Afterward, it was treated two times with 0.5% NaOH at 90 °C for 30 min to remove microbial contaminants and other impurities adsorbed on BC gels, and then continuously washed with ddH_2_O until neutrality. Finally, the dry weight of the purified BC gels was determined after drying at 70 °C overnight to a constant weight^136^. BC yield (g/L) and production rate (g/L/d) were calculated accordance with the method of Kan et al.^137,138^ using the following equations:

2 $$ {\text{BC}}\;{\text{yield}}\left( {{\text{g}}/{\text{L}}} \right) = {\text{BC}}\;{\text{dry}}\;{\text{weight}}\left( {\text{g}} \right)/{\text{Volume}}\;{\text{of}}\;{\text{production}}\;{\text{media}} $$

and

3 $$ {\text{BC production rate}}\left( {{\text{g}}/{\text{L}}/{\text{d}}} \right) = {\text{BC}}\;{\text{yield}}\left( {{\text{g}}/{\text{L}}} \right)/{\text{Tt}} $$

Tt indicates the total production time.

**Table 6:** Compositions of different media applied for BC production from *L. plantarum* AS.6.

Media	Composition (g/L)
HS	Glucose (20), yeast extract (5), peptone (5), disodium hydrogen phosphate (2.7), citric acid (1.15) and ethanol (5 ml/L)
JCP	Glucose (2.04), reducing sugars (4.03) and total carbohydrates (5.52)
ECP	Glucose (5.37), reducing sugars (7.8) and total carbohydrates (9.91)
ECPS	Glucose (5.37), reducing sugars (7.8), total carbohydrates (9.91), yeast extract (5), peptone (5), disodium hydrogen phosphate (2.7), citric acid (1.15) and ethanol (5 ml/L)

#### Pomegranate peel (POP) extract preparation

First, the pomegranate fruit was washed to remove extraneous materials, and then the peels and seeds were separated manually. The POP extract was prepared by the method described by Patel et al.^139^ with minor modifications. Briefly, the fresh POP were cut into small pieces and ground for 10 min at room temperature in a high-speed blender (800ES blender, USA). Then the dispersed POP mixture at concentration of 1:5 (solid: water) was hydrothermally extracted at 100 °C for 45 min. After cooling, the mixture was centrifuged at 15,000 rpm for 15 min at 4 °C, where the POP supernatant was collected and stored at 4 °C for further use.

#### Green synthesis of CuO-NPs

The green synthesis of CuO-NPs was performed using POP extract in accordance with the method of Ramzan et al.^140^ with little modifications. 0.5 M copper (II) nitrate trihydrate (Cu(NO_3_)_2_·3H_2_O) was added to 100 mL of POP extract. The mixture pH was adjusted to 12 with the addition of ammonia solution under constant stirring and heated at 75 °C for 5–6 h. The formation of the brown precipitate indicated the complete Cu reduction with the formation of CuO-NPs. The resulting solution was centrifuged at 4000 rpm for 15 min, where the precipitate (metal-NPs) was washed several times using ethanol and ddH_2_O, then dried in an oven for aging at 70 °C overnight. The sample was then calcinated at 450 °C for 4 h, where the resulting CuO-NPs were applied in the following experiments.

#### Preparation of BC/PVA/Ch/CuO-NPs composite

A simple solution mixing and casting approach was used to prepare the BC/PVA/Ch composite. The wet BC membrane (50 g) was cut into small pieces by using scissors and dispersed in 100 mL of ddH_2_O by using a high-speed blender (800ES blender, USA) for 15 min to form BC slurry (solution A). PVA solution at 4% (w/v) was obtained by dissolving PVA in ddH_2_O (solution B), whereas Ch solution at 1% (w/v) was obtained by using a 1% aqueous solution of acetic acid (solution C). Both solutions (B and C) were obtained by occasionally stirring at room temperature until the solutions became homogenous and viscous. Approximately 10 mL of each solution was mixed to obtain homogenous BC/PVA/Ch dispersions and hybrid composite. Finally, CuO-NPs at different concentrations (0.025, 0.050, 0.075, and 0.100 mg) were added to the 30 mL of BC/PVA/Ch dispersions with two drops of glycerol as a stabilizing agent and homogenized through ultra-sonication (750 wt, 20 KH, pulse 45, Amp 1) for 5 min. The mixture of each concentration was poured into a 6 cm petri dish and left to dry at 50 °C for 48 h. The BC/PVA/Ch membranes free from CuO-NPs were used as a control. The membranes were carefully peeled off from the plates and stored in a vacuum desiccator until further use.

### Characterization of samples

#### Instrumental characterizations

*Scanning Electron Microscopy (SEM)*. The surface structure and elemental analysis of samples were investigated by SEM (SEM, Joel GSM-6610LV, Japan). The average diameter of nanofiber was determined from different SEM images using the angle tool of ImageJ with Java 1.8.0 software (National Institute of Health (NIH), USA)^141^. *Fourier transform infrared spectroscopy (FT-IR)*: The chemical structural differences of obtained samples were analyzed by FT-IR (IR, 8400 s Shimadzu, Japan) with IR fingerprints recorded between 4000 and 400 cm^−1^ using transmittance modes. *X-ray diffraction (XRD)*: The overall crystalline phases of the obtained BC samples were determined by XRD measurement (X-ray diffractometers, Malvern Panalytical Empyrean, France). Radial scans of intensity were recorded at ambient conditions over scattering two angles ranging from 5° to 80° with a step increment of 0.02°/s. *Thermogravimetric analyzer (TGA)*: Thermal decomposition as a function of weight loss% of BC samples was performed by TGA (TGA-50, Shimadzu, Japan). Dried samples were operated under nitrogen gas at a heating rate of 10 °C/min and temperature range of 25 °C to 800 °C. *Particle size*: Particle size and zeta potential of the prepared CuO-NPs were measured using a Zeta-Sizer (Malvern, UK).

#### Physicochemical characterization of composite membranes

*Thickness measurement*: The thickness of the dry composite membranes was measured using an electronic digital micrometer (Absolute Digimatic, CD-15APX, Japan). Five random points of each composite membrane were selected for the measurements, and the average of the obtained readings was represented^142^. *Moisture contents (MC)*: The moisture contents (MC) of the resulting cellulose membranes were calculated as a percentage of their oven-dry weight by comparing their weights before and after drying at 80 °C. The MC was calculated according to the following equation

4 $$ MC\% = \left( {W_{0} - W_{1} } \right)/W_{1} $$

where *W*_*0*_ is the initial weight of samples before drying (g) and *W*_*1*_ is the weight of dried samples (g). *Composites content (CC)*: The CC was performed by the method described by Gholamali et al.^104^ with minor changes. The prepared composites were initially weighted and then socked in ddH_2_O at room temperature for 2 days at 100 rpm. At the time interval (12 h) the ddH_2_O was replaced in the sample container to remove soluble parts from the composites, after which swollen samples were taken out, dried at 50 °C, and weighted. The CC of prepared samples was calculated according to the given equation

5 $$ CC\% = W_{f} /W_{i} $$

where *W*_*i*_ and *W*_*f*_ represent the initial and final weights of the dried samples, respectively. *Swelling ratio (SR)*: The membranes SR was expressed as the ability of composite membranes to uptake any physiological fluids (water) over time. The composites membranes were initially weighed and immersed in 50 mL of ddH_2_O. After specified time intervals, samples were removed, where extra water was wiped with soft tissue paper and weighted. The SR was calculated according to Salim et al.^143^ using the following equation

6 $$ SR\% = \left( {W_{r} - W_{i} } \right)/W_{i} $$

where *W*_*i*_ is the weight of dry samples (g) and *W*_*r*_ is the swollen samples weight (g).

### Biological evaluations of BC/PVA/Ch/CuO-NPs composite membranes

#### Antimicrobial activity

##### Microbial used and growth conditions

The antimicrobial activity of the prepared membranes was assessed against nine selected pathogens, including *Escherichia coli* ATCC 25,922 (*E. coli*), *Klebsiella pneumoniae* ATCC 13,883 (*K. pneumoniae*), *Salmonella typhimurium* ATCC 14,028 (*S. typhimurium*), *Pseudomonas fluorescens* DSMZ 50,090 (*P. fluorescens*) and *Aeromonas hydrophila* NRL 914 (*A. hydrophila*) as Gram-negative bacteria*, Bacillus subtilis* ATCC6633 (*B. subtilis*)*, Staphylococcus aureus* ATCC 25,923 (*S. aureus*), and *Streptococcus mutans* ATCC 25,175 (*S. mutans*) as Gram-positive bacteria, and *Candida albicans* ATCC 10,231 (*C. albicans*) as a yeast model. The reference microbes were obtained from the American Type Culture Collection (ATCC), Deutsche Sammlung von Mikroorganismen und Zellkulturen (DSMZ) and National Reference Laboratory. The microbial strains were cultivated in a nutrient broth containing (g/L): 3 yeast extract, 5 peptone, and 5 sodium chloride and incubated at 37 °C under shaking at 200 rpm for 24 h.

##### Disk diffusion technique

The antimicrobial activity of the prepared BC/PVA/Ch/CuO-NPs composites was investigated through disk diffusion approach as described by Yahia et al.^144^, with minor modifications. In brief, 100 µl of serially diluted pathogens (10^8^ CFU/mL) was separately distributed on Petri dishes of nutrient agar media, along with disks (5 mm in diameter) of the composite membranes loaded with different concentrations of CuO-NPs. The CuO-NPs-free BC/PVA/Ch composite membrane was used as a control (negative control). The plates were incubated at 37 °C for 24 h, where the antibacterial activity was assessed by measuring the developed inhibition-zone diameter (including the membrane disk) after incubation period. All membranes were sterilized for 30 min under UV light before application to ensure aseptic conditions. All experiments were conducted in triplicate and the mean result was represented.

#### Cytotoxicity and anticancer activity

##### Cytotoxicity effects of BC/PVA/Ch/CuO-NPs composites on HSF cells

To evaluate the compatibility of the prepared BC/PVA/Ch/CuO-NPs composites membranes on normal cells, the method of MTT [3-(4,5-dimethylthiazol-2-yl)- 2,5 diphenyl tetrazolium bromide] was used as described by Mosmann et al. and Abu-Serie et al.^145,146^, respectively. In brief, the HSF (somatic normal skin cells) cell line (5.0 × 10^3^) was cultured in a sterile 24-well tissue culture plate using DMEM media (Lonza, USA) supplemented with 10% fetal bovine serum (FBS) overnight. After incubation, different discs from BC/PVA/Ch/CuO-NPs composite membranes at weights of 0.5, 1.0, 2.0, 3.0, and 4.0 mg/mL were added to cells in triplicates. After another incubation for 48 h at 37 °C, with 5% CO_2_ and 90% humidity, the cell viability was evaluated by MTT solution (0.5 mg/mL, Sigma-Aldrich). After incubation for 2–5 h, DMSO was added, and the absorbance was measured at 570 nm using a microplate reader (BMG LabTech, Germany). The value of half-maximal inhibitory concentration (IC_50_) of each prepared BC/PVA/Ch/CuO-NPs composite was determined by the GraphPad Prism 6.0 software, and compared to disc-free wells as control (100% viability).

##### Effects of BC/PVA/Ch/CuO-NPs composites on different cancer cell lines

The anticancer activity of the prepared BC/PVA/Ch/CuO-NPs composites was evaluated in vitro against three cancer cell lines, including Caco-2 (colon carcinoma), HepG-2 (hepatoma), and MDA (breast carcinoma). Both Caco-2 and MDA cell lines were maintained in a supplemented DMEM media with 10 FBS, while HepG-2 cells were maintained in RPMI-1640 (Lonza, USA) supplemented with 5% FBS. The effect of BC/PVA/Ch/CuO-NPs composites membranes on the morphology of all tested tumor cells at IC_50_ doses was investigated by phase-contrast microscopy (Olympus, Germany) as compared to untreated control cells. The three cancer cell lines (5.0 × 10^3^ cells/well) were cultured in sterile 24-well tissue culture plates for 24 h. Discs of different weights (0.5, 1.0, 2.0, 3.0, and 4.0 mg/mL) from each BC/PVA/Ch/CuO-NPs composites were added separately to each cell line in triplicates. After 48 h of incubation at 5% CO_2_, the cytotoxic effect was evaluated through the MTT assay as described above. Also, the IC_50_ value of each BC/PVA/Ch/CuO-NPs composite was determined by the GraphPad Prism 6.0 software, and values of the selectivity index (SI) that was defined as the ratio of the IC_50_ on normal human cells (HSF) versus the IC_50_ value of each cancer cell line were previously estimated^147,148^. Furthermore, the untreated cells (disc-free wells) were considered a negative reference.

#### Larvicidal activity

##### Mosquito culture

The larvae of *A aegypti* were reared and collected using the method described by Kamaraj et al.^149^. The larvae of *A aegypti* were collected from water bottles, tanks, containers, and small water courses. The collected larvae were kept at 27 ± 2 °C with relative humidity of 80% to10% during a 12 h light and dark photoperiod cycle. In the laboratory, the larvae were fed a dog-biscuit and a brewer's yeast powder mixture in a 3:1 ratio. After five days, adult male mosquitoes were fed a 10% sucrose solution. For 2–3 h, emerging female mosquitoes obtained a blood meal from a white albino rat to produce eggs.

##### Mosquito larvicidal and pupicidal activity evaluation

Larvicidal activity was determined in accordance with the method of World Health Organization (WHO)^150^, with minor modifications^151^. After exposing to BC/PVA/Ch/ composite membranes at various concentrations of CuO-NPs, the larvicidal and pupicidal assays were conducted. Batches of 10 fourth instar larvae or pupae were placed in 50 mL of test media containing a specific concentration of BC/PVA/Ch/ CuO-NPs or tap water alone (control). In the laboratory, all containers were kept at room temperature with a naturally occurring photoperiod. As previously described by Koodalingam et al.^152,153^, the mortality of larvae and pupae in control and test media was recorded after 12 and 24 h of exposure. Dead larvae and pupae were immediately removed from the control and test media.

##### Statistical analysis

All experiments were conducted in triplicates and means ± standard deviation were represented. The statistical significance between data sets was determined through CoStat software using the least significant difference analysis of variance (ANOVA) at the 0.05 level of probability. The statistical significance was indicated with letters in ascending order where the statistical significance of letter a > b > c, etc. within the same column at *p* < 0.05.

## Data Availability

The datasets used and/or analyzed during the current study are available from the corresponding author on reasonable request.
